# A Single-center Comparison of Surgical Outcomes following Prepectoral and Subpectoral Implant-based Breast Reconstruction

**DOI:** 10.1097/GOX.0000000000005880

**Published:** 2024-06-10

**Authors:** Karie Villanueva, Harsh Patel, Durga Ghosh, Alexandra Klomhaus, Ginger Slack, Jaco Festekjian, Andrew Da Lio, Charles Tseng

**Affiliations:** From the * Department of Surgery, Division of Plastic and Reconstructive Surgery, University of California, Los Angeles David Geffen School of Medicine, Los Angeles, Calif.; † Los Angeles David Geffen School of Medicine, University of California, Los Angeles, Calif.; ‡ Department of Medicine Statistics Core, David Geffen School of Medicine, University of California Los Angeles, Los Angeles, Calif.

## Abstract

**Background:**

Prepectoral implant placement continues to gain widespread acceptance as a safe and effective option for breast reconstruction. Current literature demonstrates comparable rates of complications and revisions between prepectoral and subpectoral placement; however, these studies are underpowered and lack long-term follow-up.

**Methods:**

We performed a retrospective cohort study of patients who underwent immediate two-staged tissue expander or direct-to-implant breast reconstruction at a single center from January 2017 to March 2021. Cases were divided into prepectoral and subpectoral cohorts. The primary outcomes were postoperative complications, aesthetic deformities, and secondary revisions. Descriptive statistics and multivariable regression models were performed to compare the demographic characteristics and outcomes between the two cohorts.

**Results:**

We identified 996 breasts (570 patients), which were divided into prepectoral (391 breasts) and subpectoral (605 breasts) cohorts. There was a higher rate of complications (*P* < 0.001) and aesthetic deformities (*P* = 0.02) with prepectoral breast reconstruction. Secondary revisions were comparable between the two cohorts. Multivariable regression analysis confirmed that prepectoral reconstruction was associated with an increased risk of complications (odds ratio 2.39, *P* < 0.001) and aesthetic deformities (odds ratio 1.62, *P* = 0.003).

**Conclusions:**

This study evaluated outcomes in patients undergoing prepectoral or subpectoral breast reconstruction from a single center with long-term follow-up. Prepectoral placement was shown to have an inferior complication and aesthetic profile compared with subpectoral placement, with no difference in secondary revisions. These findings require validation with a well-designed randomized controlled trial to establish best practice for implant-based breast reconstruction.

**Takeaways**

**Question:** Does prepectoral implant-based breast reconstruction (IBBR) have a superior complication profile, aesthetic outcomes, and/or secondary revision rate compared with subpectoral IBBR?

**Findings:** Our retrospective review demonstrates a higher rate of complications and aesthetic deformities with prepectoral reconstruction. However, secondary revisions were comparable between the two groups.

**Meaning:** Subpectoral IBBR may have a favorable complication and aesthetic profile compared with prepectoral IBBR.

## INTRODUCTION

Implant-based breast reconstruction (IBBR) is the most common method for breast reconstruction with steadily increasing rates over the past several years.^1,2^ The ideal anatomical placement of tissue expanders (TEs) and implants has been the subject of discussion and has evolved over time. Traditionally, subpectoral IBBR was considered standard of care due to prior concerns regarding complications with prepectoral implant placement, especially in the setting of postmastectomy radiation (PMRT). PMRT presents a unique challenge because radiation increases the risk for implant exposure, implant loss, and capsular contracture.^3–6^ Subpectoral reconstruction was believed to reduce such risks by providing an additional layer of vascularized tissue over the implant.

More recently, prepectoral IBBR has gained widespread popularity as an alternative technique for breast reconstruction. Recent advances in surgical techniques have stimulated this renewed interest, including refinements in mastectomy techniques and inclusion of acellular dermal matrix (ADM).^7–10^ Some studies have shown protective effects against radiation, such as reconstructive failure, capsular contracture, and muscle fibrosis.^5,11^ Other studies have also demonstrated shorter postoperative recovery time, reduced postoperative visits, decreased pain scores, and improved aesthetics.^12–14^

Another consideration is secondary procedures following IBBR. Fischer et al^15^ retrospectively examined healthcare resource utilization after IBBR in the United States using state-level surgery databases. They found that the implant cohort had the highest number of revisions compared with other methods of breast reconstruction.^15^ Although the rate of procedures may be influenced by patient selection and surgeon preference, there is no clear understanding regarding the predictors of revisions required for completion of reconstruction.^16–19^ Furthermore, there have not been many comprehensive studies that compare rates of secondary revision procedures after prepectoral and subpectoral reconstruction.

Altogether, prepectoral IBBR is a revisited technique with a paucity of studies yielding long-term outcomes. Published studies have focused on comparing complications, and less frequently, aesthetic outcomes between prepectoral and subpectoral reconstruction. The aim of this study was to provide long-term data comparing postoperative complications, aesthetic outcomes, and rates of secondary revision procedures between prepectoral and subpectoral IBBR at a single institution.

## METHODS

### Patient Identification and Data Collection

A retrospective study was performed of patients who underwent breast reconstruction at University of California, Los Angeles Medical Center from January 2017 to March 2021 with follow-up ending on January 2023. We began collecting data from 2017 because this was the period our surgeons began performing prepectoral reconstruction. The database was generated using Epic electronic medical records (Epic Systems Corp., Wis.) using current procedural terminology codes 19357 and 15777. A total of 1761 patients were identified. Of them, 1,191 patients were then excluded based on exclusion criteria and miscoding. Within our final cohort, 570 charts were reviewed. It was restricted to patients who underwent mastectomy for breast cancer or cancer prophylaxis. Patients who underwent immediate prepectoral or subpectoral breast reconstruction with ADM and TEs or direct to implant (DTI) were included. Those who underwent delayed IBBR or immediate autologous-based breast reconstruction were excluded. Each breast was counted once and entered in the study at the time of expander or implant placement. The type of mastectomy (ie, skin sparing or nipple sparing) and the final stage of reconstruction were recorded.

Patient information was then manually recorded by author K.V. and H.P. and validated by author C.T., including patient age, body mass index (BMI) at the time of the index surgery (categorized into five groups according to World Health Organization classification), presence of diabetes, smoking history (ie, current, former, or nonsmoker), initial cancer stage, previous breast surgery (eg, prior lumpectomy/radiation, reduction mammoplasty, mastopexy, augmentation mammoplasty, skin flap, or nipple delay), radiation therapy (ie, prior history or adjuvant therapy), and chemotherapy (ie, neoadjuvant or adjuvant therapy). Complications were recorded, which included any complication, seroma, infection, hematoma, skin and nipple necrosis, wound dehiscence, soft-tissue infection, reoperation, and implant/expander removal. Complications were further stratified into major or minor complications. Major complications were defined as those requiring surgical intervention. Minor complications were defined as those managed with conservative measures, such as aspiration of seroma in clinic, treatment of soft-tissue infections with oral or intravenous antibiotics, or local wound care for skin flap and nipple necrosis. We looked at complications per breast because each breast had its own individual risk factors. For example, a patient with unilateral breast cancer who undergoes bilateral mastectomies may receive radiation to one breast, increasing its risk of developing complications compared with the noncancer breast. Aesthetic deformities were recorded, including capsular contracture, animation deformity, and rippling. Finally, unplanned secondary revisions were documented, defined as revisions performed following implant exchange in the TE group and DTI reconstruction, including autologous fat grafting, breast mound revisions, and conversion to autologous flaps.

### Surgical Technique

Prepectoral and subpectoral IBBRs were performed by six plastic surgeons. The mastectomies were performed by four breast surgeons. Breast reconstruction options were discussed with the patient preoperatively; however, the decision to proceed with prepectoral versus subpectoral expander or implant placement was made based on patient preference and intraoperatively following qualitative assessment (eg, palpation and visualization) of mastectomy skin flap perfusion. ADM (AlloDerm, LifeCell/Allergan, Irvine, Calif.) was used in all patients. TEs or silicone implants (Allergan, Mentor, or Sientra) were used in each case. Methods for ADM placement were based on surgeon preference. For the prepectoral cohort, one surgeon inserted a sheet of ADM into the subcutaneous pocket and sutured the ADM along the perimeter of the chest wall in accordance with the dimensions of the TE or implant. The remaining five surgeons wrapped the TE or implant with ADM and secured it with posterior spanning sutures. For the subpectoral cohort, a sheet of ADM was fashioned into a sling where it was sutured along the leading edge of the pectoralis muscle to the inframammary fold and laterally to close the expander pocket. All patients received 24 hours of perioperative antibiotics followed by a 7-day course or postoperative antibiotics. One or two drains were placed in each mastectomy pocket. For TE cases, expansion with saline resumed approximately 2 weeks postoperatively. TE exchange with permanent implants or conversion to autologous flap was usually performed at least 6 months after completion of radiation or 1 month after final expansion in nonradiated cases. Additional secondary revision procedures were performed based on patient concerns and surgeon discretion.

### Statistical Analysis

We present descriptive statistics, including means and SDs for continuous variables and counts and frequencies for categorical variables, for both demographic and clinical characteristics. We stratified results by prepectoral and subpectoral cohorts. To test for differences in demographic and clinical characteristics between cohorts, we used chi-square tests (or Fisher exact tests) and Wilcoxon rank-sum tests for categorical and continuous variables, respectively. Additionally, complications from expander or implant placement, aesthetic deformities, and secondary revision procedures were evaluated across surgical approaches, and differences between the two approaches were assessed using chi-square tests. A subgroup analysis was performed to compare outcomes in patients who received PMRT.

Multivariable logistic regression analyses were used to examine the effect of surgical technique on developing any complication, aesthetic deformity, and undergoing an unplanned secondary procedure. Covariates were selected based on clinical relevancy irrespective of their statistical significance, including specific patient characteristics, surgical technique, and medical treatment. We present parameter estimates and standard errors, odds ratios (ORs), confidence intervals, and associated *P* values. Statistical analysis occurred at the level of the breast. All *P* values were two-sided, with a *P* value of less than 0.05 considered statistically significant. All statistical analyses were performed using SAS 9.4 (SAS Institute, Cary, N.C.).

## RESULTS

Over the study period, a total of 996 breasts from 570 patients were identified. Of them, 391 cases (39.26%) underwent prepectoral placement, and the remaining 605 cases (60.74%) underwent subpectoral placement. The mean age of the subpectoral and prepectoral cohorts were 49 and 47 years, respectively. Those in the prepectoral group had slightly higher BMI compared with the subpectoral group (24.77 versus 23.90, *P* = 0.03). Table 1 shows post host analyses and individual comparisons of patient demographics and oncologic characteristics among prepectoral and subpectoral cohorts. There was a significantly higher number of former smokers and smokers in the subpectoral group compared with prepectoral group (*P* < 0.001). There were no significant differences in rates of diabetes. With respect to oncologic characteristics, including cancer stage, treatment with preoperative and postoperative radiation, and mastectomy type, there were no significant differences between the two groups. However, a higher number of prepectoral cases received neoadjuvant (104 cases, 26.6%) and adjuvant (148 cases, 37.8%) chemotherapy compared with subpectoral cases, which was statistically significant (Table 1).

**Table 1.** Patient and Surgical Characteristics

	Total	Prepectoral	Subpectoral	*P * *
No. breasts	996	391	605	
No. patients	570	231	339	
Age (at surgery), y, mean (SD)	48.6 (11.5)	47.29 (12.24)	49.47 (10.85)	0.0007†
Age (categorical), y				<0.0001†
≤40	237 (23.8)	130 (33.3)	107 (17.7)	
>40 and ≤54	496 (49.8)	158 (40.4)	338 (55.9)	
>54	263 (26.4)	103 (26.3)	160 (26.5)	
BMI, kg/m^2^, mean (SD)	24.24 (4.51)	24.77 (4.76)	23.90 (4.32)	0.0272†
BMI (categorical), kg/m^2^				<0.0001†
≤18.5	50 (5.0)	10 (2.6)	40 (6.6)	
>18.5 and ≤24.9	570 (57.2)	232 (59.3)	338 (55.9)	
>24.9 and ≤29.9	254 (25.0)	74 (18.9)	180 (29.8)	
>30	122 (12.3)	75 (19.2)	47 (7.8)	
Smoking status				0.0008†
None	843 (84.6)	351 (89.8)	492 (81.3)	
Active	10 (1.0)	1 (0.3)	9 (1.5)	
Former	143 (14.4)	39 (10.0)	104 (17.2)	
Diabetes				0.3432
Yes	34 (3.4)	16 (4.1)	18 (3.0)	
Cancer stage				
0	169 (17.0)	64 (16.4)	105 (17.4)	0.8723
I	295 (29.6)	118 (30.2)	177 (29.3)	
II	227 (22.8)	88 (22.5)	139 (23.0)	
III	109 (10.9)	48 (12.3)	61 (10.1)	
IV	24 (2.4)	10 (2.6)	14 (2.3)	
Genetic carriers (eg, BRCA)	172 (17.3)	63 (16.1)	109 (18.0)	
Neoadjuvant chemotherapy				0.0406†
Yes	231 (23.2)	104 (26.6)	127 (21.0)	
Adjuvant chemotherapy				<0.0001†
Yes	274 (27.5)	148 (37.8)	126 (20.8)	
Prior radiation				0.3143
Yes	15 (1.5)	4 (1.0)	11 (1.8)	
Adjuvant radiation				0.0791
Yes	163 (16.4)	74 (18.9)	89 (14.7)	
Mastectomy type				0.0574
Nipple sparing	374 (37.6)	161 (41.2)	213 (35.2)	
Skin sparing	622 (62.4)	230 (58.8)	392 (64.8)	
Reconstruction type				0.0004†
Tissue expander (2-stage)	953 (95.5)	363 (92.8)	590 (97.2)	
Direct-to-implant (1-stage)	45 (4.5)	28 (7.2)	17 (2.8)	
Laterality				0.0031†
Unilateral	145 (14.6)	73 (18.7)	72 (11.9)	
Bilateral	851 (85.4)	318 (81.3)	533 (88.1)	
Initial fill volume, mL	255.73	291.23	232.79	<0.0001†
Expander exchange to implant				<0.0001†
Yes	862 (86.6)	311 (79.5)	551 (91.1)	
Completed reconstruction				0.2402
Yes	968 (97.2)	383 (98.0)	585 (96.7)	
Prior breast surgery				<0.0001†
Yes	267 (26.8)	62 (15.9)	205 (33.9)	
No. breasts‡	267	62	205	
Lumpectomy/radiation				0.0519
Yes	58 (21.7)	19 (30.6)	39 (19.0)	
Breast augmentation				<0.0001†
Yes	134 (50.2)	14 (22.6)	120 (58.5)	
Breast reduction or mastopexy				0.3278
Yes	53 (19.8)	15 (24.2)	38 (18.5)	
Skin flap or nipple delay				0.2628
Yes	96 (36.0)	26 (41.9)	70 (34.1)	

Previous breast surgery indicates lumpectomy, breast augmentation, breast reduction or mastopexy, skin flap or nipple delay.

* A *P* value from chi-square tests (or Fisher exact, when applicable) for categorical variables and Wilcoxon tests for continuous variables.

† Values are statistically significant.

‡ Lumpectomy, breast augmentation, breast reduction or mastopexy, and skin flap or nipple delay were all calculated among the subsample of breasts where a prior breast surgery was indicated.

BMI, body mass index.

DTI reconstructions were performed in 45 cases (4.5%), whereas the remaining 953 cases (95.5%) underwent two-stage TE reconstruction. When TEs were used, the initial volume of inflation was significantly higher in the prepectoral group compared with the subpectoral group (291 versus 232 mL, *P* < 0.001). The rate of exchange to permanent implants was significantly higher in the subpectoral group (91.1%) compared with the prepectoral group (79.5%). There was a significantly higher number of patients who underwent prior breast surgery (33.9%), including breast augmentation (19.8%) in the subpectoral group compared with the prepectoral group. There was also a slightly higher number of bilateral cases performed in the subpectoral group (88.1% versus 81.3%). The mean follow-up periods were 28 and 34 months for the prepectoral and subpectoral TE groups, and 8 and 21 months in the prepectoral and subpectoral DTI groups, respectively (Table 1).

**Fig. 1.**
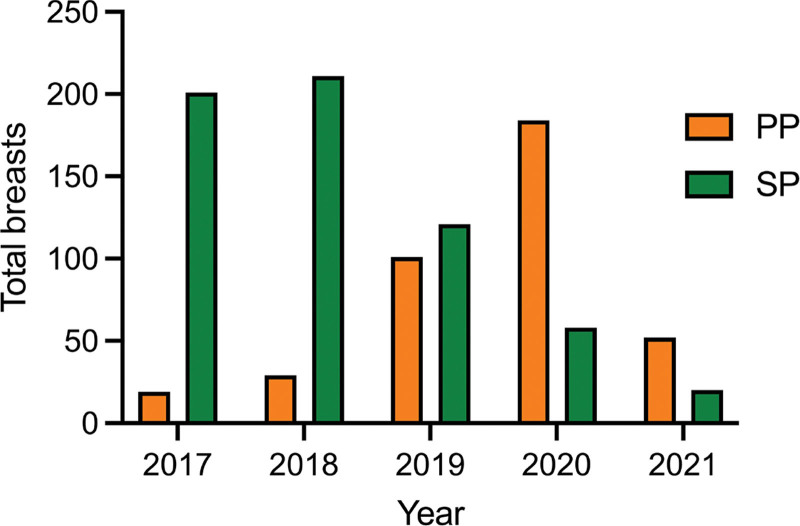
Annual rate of implant-based breast reconstruction by surgical approach (ie, prepectoral and subpectoral). PP, prepectoral IBBR; SP, subpectoral IBBR.

**Fig. 2.**
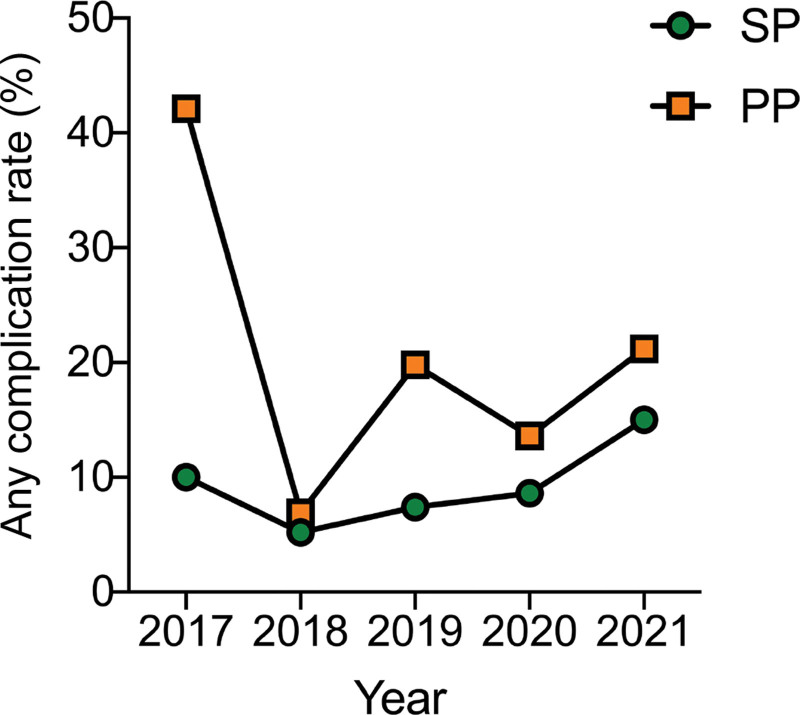
Annual complication rate of implant-based breast reconstruction by surgical approach (ie, prepectoral and subpectoral). PP, prepectoral IBBR; SP, subpectoral IBBR.

The overall complication rate was 11.4%. In univariate analysis the individuals in the prepectoral group (17.1%) had a higher rate of complications compared with the subpectoral group (7.8%). The prepectoral group had significantly higher major complications (13.3%), minor complications (3.8%), extrusion (1.5%), skin or nipple necrosis (6.1%), seroma (3.3%), wound dehiscence (1.3%), soft-tissue infection (6.6%), and need for reoperation (13.3%) (Table 2). When reviewing the number of cases performed annually, there was a higher number of subpectoral compared with prepectoral cases between 2017 and 2019. This trend gradually reversed, and by 2020, the majority of cases were performed using the prepectoral approach (Fig. 1). Overall, the annual rate of complications was higher for prepectoral compared with subpectoral IBBR. However, although the complication rates were initially high for prepectoral IBBR (42% in 2017), the rates decreased to a rate similar to that of subpectoral IBBR (Fig. 2).

**Table 2.** Complications after Expander and Implant Surgery

	Total	Prepectoral	Subpectoral	*P * *
No. breasts	996	391	605	
Any complications				<0.0001†
Yes	114 (11.4)	67 (17.1)	47 (7.8)	
Major complications				0.0002†
Yes	90 (9.0)	52 (13.3)	38 (6.3)	
Minor complications				0.0183†
Yes	24 (2.4)	15 (3.8)	9 (1.5)	
Extrusion				0.0377†
Yes	8 (0.8)	6 (1.5)	2 (0.3)	
Skin or nipple necrosis				0.0003†
Yes	35 (3.5)	24 (6.1)	11 (1.8)	
Seroma				0.0172†
Yes	20 (2.0)	13 (3.3)	7 (1.2)	
Hematoma				0.6492
Yes	18 (1.8)	8 (2.0)	10 (10.6)	
Wound dehiscence				0.0372†
Yes	6 (0.6)	5 (1.3)	1 (0.2)	
Soft-tissue infection				0.0424†
Yes	49 (4.9)	26 (6.6)	23 (3.8)	
Reoperation				0.0002†
Yes	90 (9.0)	52 (13.3)	38 (6.3)	
Expander/implant removal				0.1333
Yes	35 (3.5)	18 (4.6)	17 (2.8)	

* A *P* value from chi-square tests (or Fisher exact, when applicable) for categorical variables and Wilcoxon tests for continuous variables.

† Values are statistically significant.

With regard to aesthetic deformities, the prepectoral group had a higher rate of overall deformities (26.6%), including rippling (22%). However, the subpectoral group had a higher rate of animation deformity (2.8%). There were no significant differences in rates of capsular contracture between the two cohorts (Table 3). When examining secondary surgery, the overall rate was 27.3% for unplanned secondary revisions. In univariate analysis, there were no significant differences in fat grafting and breast mound revisions between the two cohorts. However, there was a higher number of individuals who underwent conversion to autologous flaps in the prepectoral cohort (11%) (Table 4).

**Table 3.** Aesthetic Deformities after Expander and Implant Surgery

	Total	Prepectoral	Subpectoral	*P * *
No. breasts	996	391	605	
Aesthetic deformities				0.0150†
Yes	225 (22.6)	104 (26.6)	121 (20.0)	
Rippling				<0.0001†
Yes	160 (16.1)	86 (22.0)	74 (12.1)	
Animation deformity				0.0096†
Yes	17 (1.7)	0 (0)	17 (2.8)	
Capsular contracture				0.5531
Yes	68 (6.8)	29 (7.4)	39 (6.4)	

* A *P* value from chi-square tests (or Fisher exact, when applicable) for categorical variables and Wilcoxon tests for continuous variables.

† Values are statistically significant.

**Table 4.** Unplanned Secondary Revision Surgery after Expander and Implant Surgery

	Total	Prepectoral	Subpectoral	*P * *
No. breasts	996	391	605	
Any unplanned revisions				0.2240†
Yes	272 (27.3)	94 (24.0)	178 (29.4)	
Fat grafting				0.1380
Yes	175 (17.6)	60 (15.3)	115 (19.0)	
Breast mound revision				0.7692
Yes	139 (14.0)	53 (13.5)	86 (14.2)	
Conversion to autologous flaps				0.0001‡
Yes	71 (7.1)	43 (11.0)	28 (4.6)	

* A *P* value from chi-square tests (or Fisher exact, when applicable) for categorical variables and Wilcoxon tests for continuous variables.

† A *P* value excludes patients who did not complete reconstruction.

‡ Values are statistically significant.

All three outcomes were also compared between the two cohorts in the setting of adjuvant radiation. Of the irradiated breasts, there were no significant differences in any complications between the prepectoral and subpectoral cohorts (Table 5). The prepectoral group had a higher rate of aesthetic deformities (28.4%) compared with the subpectoral group, including rippling (20.3%, Table 6). Like the entire cohort, there was a higher rate of conversion to autologous flaps in the prepectoral group (25.7%), and no significant differences in fat grafting and breast revisions between the two groups (Table 7).

**Table 5.** Complications after Expander and Implant Surgery in the Setting of Postmastectomy Radiation

	Total	Prepectoral	Subpectoral	*P * *
No. breasts	163	74	89	
Any complications				0.5598
Yes	32 (19.6)	16 (21.6)	16 (18.0)	
Major complications				0.4609
Yes	27 (16.6)	14 (18.9)	13 (14.6)	
Minor complications				1.0000
Yes	5 (3.1)	2 (2.7)	3 (3.4)	
Extrusion				0.5910
Yes	3 (1.8)	2 (2.7)	1 (1.1)	
Skin or nipple necrosis				0.0803
Yes	9 (5.5)	7 (9.5)	2 (2.2)	
Seroma				0.1267
Yes	4 (2.4)	0 (0.0)	4 (4.5)	
Hematoma				1.0000
Yes	5 (3.1)	2 (2.7)	3 (3.4)	
Wound dehiscence				0.4540
Yes	1 (0.6)	1 (1.3)	0 (0.0)	
Infection				0.7118
Yes	17 (10.4)	7 (9.5)	10 (11.2)	
Reoperation				0.4609
Yes	27 (16.6)	14 (18.9)	13 (14.6)	
Expander or implant removal				0.7873
Yes	12 (7.4)	5 (6.8)	7 (7.9)	

* A *P* value from chi-square tests (or Fisher exact, when applicable) for categorical variables and Wilcoxon tests for continuous variables.

**Table 6.** Aesthetic Deformities after Expander and Implant Surgery in the Setting of Postmastectomy Radiation

	Total	Prepectoral	Subpectoral	*P * *
No. breasts	163	74	89	
Aesthetic deformities				0.0185†
Yes	33 (2.2)	21 (28.4)	12 (13.5)	
Rippling				0.0103†
Yes	21 (12.9)	15 (20.3)	6 (6.7)	
Animation deformity				1.0000
Yes	1 (0.6)	0 (0.0)	1 (1.1)	
Capsular contracture				0.1377
Yes	14 (8.6)	9 (12.2)	5 (5.6)	

* A *P* value from chi-square tests (or Fisher exact, when applicable) for categorical variables and Wilcoxon tests for continuous variables.

† Values are statistically significant.

**Table 7.** Unplanned Secondary Revision Surgery after Expander and Implant Surgery in the Setting of Postmastectomy Radiation

	Total	Prepectoral	Subpectoral	*P * *
No. breasts	163	74	89	
Any unplanned revisions				0.2511†
Yes	33 (20.2)	11 (14.9)	22 (24.7)	
Fat grafting				0.7590
Yes	19 (11.7)	8 (10.8)	11 (12.4)	
Breast mound revision				0.9695
Yes	20 (12.3)	9 (12.2)	11 (12.4)	
Conversion to autologous flap				0.0483‡
Yes	31 (19.0)	19 (25.7)	12 (13.5)	

* A *P* value from chi-square tests (or Fisher exact, when applicable) for categorical variables and Wilcoxon tests for continuous variables.

† A *P* value excludes patients who did not complete reconstruction.

‡ Values are statistically significant.

To confirm our findings, a multivariable regression analysis was performed exploring the association of expander and implant plane and other clinical variables with the development of any complications, aesthetic deformities, and unplanned secondary revisions. Covariates that were found to be clinically relevant included age, BMI, smoking status, diabetes status, adjuvant radiation, prior breast surgery, cancer stage, and expander or implant placement. These variables were used to perform stepwise multivariable logistic regression analyses. Individuals who underwent prepectoral expander or implant placement were 2.39 times more likely to develop a complication compared with those who underwent subpectoral placement. Similarly, increased age (OR 1.02, *P* = 0.02), higher BMI (OR 1.06, *P* = 0.004), and adjuvant radiation (OR 1.83, *P* = 0.02) demonstrated significant associations with complications (Table 8). When looking at aesthetic outcomes, prepectoral placement was independently associated with increased risk of deformities (OR 1.61, *P* = 0.003). Having a higher BMI (OR 0.95, *P* = 0.008) and advanced cancer stage (OR 0.66, *P* = 0.01) was associated with decreased risks of developing an aesthetic deformity (Table 9). Finally, the regression model demonstrated no independent associations with unplanned surgical revisions between the two cohorts (Table 10).

**Table 8.** Multivariable Regression Analysis for Occurrence of Any Complication between Prepectoral and Subpectoral Groups

Variable	Reference Group	Est. (SE)	OR	95% CI	*P*
Any complication					
Age at time of surgery		0.02 (0.01)	1.02	(1.00–1.04)	0.0181*
BMI, kg/m^2^		0.06 (0.02)	1.06	(1.02–1.11)	0.0044*
Smoker (yes/former)	Never	0.30 (0.28)	1.36	(0.79–2.34)	0.2747
Diabetes (yes)	No	0.66 (0.45)	1.93	(0.81–4.62)	0.1402
Adjuvant radiation (yes)	No	0.60 (0.25)	1.83	(1.11–3.00)	0.0173*
Prior breast surgery (yes)	No	−0.04 (0.25)	0.96	(0.58–1.57)	0.8633
Cancer stage (I, II, III, IV)	0/g.c.	0.28 (0.25)	1.32	(0.80–2.17)	0.2722
Group (prepectoral)	Subpectoral	0.87 (0.22)	2.39	(1.57–3.66)	<0.0001*

* Values are statistically significant.

**Table 9.** Multivariable Regression Analysis for Occurrence of Aesthetic Deformities between Prepectoral and Subpectoral Groups

Variable	Reference Group	Est. (SE)	OR	95% CI	*P*
Aesthetic deformities					
Age at time of surgery		−0.01 (0.01)	0.99	(0.98–1.01)	0.3797
BMI		−0.05 (0.02)	0.95	(0.92–0.99)	0.0077*
Smoker (yes/former)	Never	0.07 (0.22)	1.08	(0.70–1.65)	0.7425
Diabetes (yes)	No	0.18 (0.45)	1.20	(0.50–2.90)	0.6869
Adjuvant radiation (yes)	No	0.03 (0.23)	1.03	(0.66–1.61)	0.8848
Prior breast surgery (yes)	No	0.31 (0.18)	1.36	(0.97–1.92)	0.0771*
Cancer stage (I, II, III, IV)	0/g.c.	−0.42 (0.17)	0.66	(0.47–0.92)	0.0131*
Group (prepectoral)	Subpectoral	0.48 (0.16)	1.61	(1.18–2.21)	0.0031*

* Values are statistically significant.

CI, confidence interval; OR, odds ratio.

**Table 10.** Multivariable Regression Analysis for Occurrence of Unplanned Secondary Procedures between Prepectoral and Subpectoral Groups

Variable	Reference Group	Est. (SE)	OR	95% CI	*P*
Unplanned secondary procedures					
Age at time of surgery		−0.01 (0.01)	0.99	(0.98–1.00)	0.1416
BMI, kg/m^2^		0.01 (0.02)	1.01	(0.98–1.04)	0.5914
Smoker (yes/former)	Never	0.02 (0.20)	1.02	(0.69–1.52)	0.9173
Diabetes (yes)	No	0.52 (0.41)	1.68	(0.76–3.73)	0.2041
Adjuvant radiation (yes)	No	−0.26 (0.22)	0.77	(0.49–1.19)	0.2389
Prior breast surgery (yes)	No	0.25 (0.16)	1.28	(0.93–1.77)	0.1297
Cancer stage (I, II, III, IV)	0/g.c.	0.05 (0.16)	1.05	(0.77–1.44)	0.7452
Group (prepectoral)	Subpectoral	−0.16 (0.16)	0.85	(0.63–1.15)	0.2928

## DISCUSSION

Prior studies have demonstrated comparable complication rates between prepectoral and subpectoral IBBR.^20–27^ In a retrospective review of 294 breasts, Bettinger et al^28^ had shown similar complication rates between prepectoral (10.2%) and subpectoral cohorts (9.5%). Comparable complication rates were demonstrated in another retrospective review: 21.5% in the subpectoral and 22.4% in the prepectoral groups.^7^ The results presented in our study differ from previous studies. There were overall higher complication rates with prepectoral IBBR compared with subpectoral IBBR, including major and minor complications. One of the most striking outcomes of the individual complications was the higher rates of skin or nipple necrosis and extrusion in the prepectoral group. This may be explained by higher initial expander volumes compared with the subpectoral group, as evidenced by prior studies.^14^ After the initial review of the annual incidence of complications, an associated learning curve may explain the higher complication rates observed initially in this study. It does seem, as surgeons became experienced with the prepectoral approach, that the complication rates were similar between the two methods.

Traditionally, PMRT was believed to have an unfavorable complication profile and result in poor aesthetic outcomes for those who undergo IBBR.^3–5,8,29^ Subpectoral reconstruction was believed to be more favorable in the setting of PMRT. However, there have been reported higher rates of capsular contracture and animation deformity from muscle retraction and fibrosis in some studies.^1^ Since its resurgence, there have been several reports on prepectoral reconstruction and PMRT. Elswick et al^4^ demonstrated no association between PMRT and complication rates following prepectoral reconstruction. However, studies comparing outcomes between prepectoral and subpectoral reconstruction in patients undergoing PMRT are limited. A meta-analysis of four studies including 390 breasts demonstrated no statistical difference of overall complications between the two cohorts.^6^ Using historical data for comparison, a subgroup analysis was performed between the two cohorts in the present study and demonstrated no significant differences in complications in the setting of PMRT.

Another important element to consider is the aesthetic outcome following IBBR. Recent literature appears to corroborate a role for prepectoral implant placement in achieving more natural breast shape and reducing capsular contracture.^11,13,30,31^ However, there is a theoretical increased risk of implant visibility and rippling. In our study, prepectoral placement was associated with a higher rate of deformities even in the setting of PMRT. As expected, implant rippling was higher in the prepectoral cohort due to the loss of additional layer of coverage between the implant and skin that the muscle provides. Such observation may also be due to an overall lower BMI seen within the study cohort.

Secondary revisions have been an integral part of breast reconstruction. From the Mastectomy Reconstruction Outcomes Consortium, 40% of breast reconstruction cases underwent revision procedures.^19^ There is no clear understanding of the factors that influence surgeons to perform secondary procedures. Losken et al^18^ described an average of four procedures to achieve a satisfactory reconstruction with higher rates in patients who received radiation therapy. Notably, several authors have reported fat grafting as an adjunct to improve aesthetic outcomes in prepectoral patients, by reduce implant visibility/rippling and camouflaging the upper pole.^11,31^ Our study, however, did not demonstrate implant plane as an independent risk factor for fat grafting. There was a significantly higher number of prepectoral cases that underwent conversion to autologous flaps. Some notable factors may explain this outcome: (1) patients’ desires to achieve an optimal cosmetic outcome to correct deformities following complications, such as extrusion, skin flap necrosis, and capsular contracture, and (2) surgeon’s preference, as some placed prepectoral devices to delay autologous reconstruction until completion of adjuvant therapy.

To the best of our knowledge, this study is one of the largest retrospective cohort studies comparing surgical and aesthetic outcomes between prepectoral and subpectoral IBBR with long-term follow-up. Most patients completed their reconstruction even in the setting of PMRT. Our results may add valuable data to the current literature on this topic. It may also facilitate preoperative patient selection and counseling during patient discussions regarding reconstructive options. Although our study suggests that patients who undergo subpectoral reconstruction may have fewer complications and aesthetic deformities overall, we cannot definitively say the prepectoral approach is an inferior technique without prospective, randomized clinical trials. It is important to note that our complication rates for prepectoral IBBR seem to be lower than those reported in recent literature.^6,7,20,26,28^ We have shown that it may still be a reasonable option for those who require PMRT and want to avoid animation deformity, and for use as a bridging method towards autologous reconstruction. Prepectoral reconstruction may also have comparable rates of revisions to the subpectoral approach.

The limitations of the current study include its nonrandomized and retrospective nature. There was also a size discrepancy between the two cohorts, with more patients in the subpectoral cohort. This can be explained by the fact that there were fewer surgeons performing prepectoral reconstruction in 2017 to 2019 with the majority performing subpectoral reconstruction during this period. The inclusion of multiple plastic and breast surgeons should be mentioned, as variations in techniques may affect the outcomes of the reconstruction performed. In some institutions, including those in Europe, it is common practice for patients to receive their oncologic and reconstructive surgery from a single surgeon. Piper et al^32^ observed lower rates of mastectomy skin flap necrosis and higher patient satisfaction for those who underwent mastectomies and reconstruction by a single-surgeon compared with the dual-surgeon approach. Furthermore, a comparison between DTI versus two-stage reconstruction was not possible in this study due to low sample size in the DTI group. Finally, our analysis focused on aesthetic parameters as observed by the plastic surgeon. Further qualitative studies that include patient-reported outcome measures may be important to explore patients’ perception of each reconstructive technique. Such research may further identify factors that influence patient’s decision-making to undergo IBBR and associated secondary procedures.

## CONCLUSIONS

We performed a large retrospective review of cases evaluating complication profiles, aesthetic outcomes, and secondary procedure rates in patients undergoing prepectoral and subpectoral breast reconstruction at a single institution. The prepectoral group was independently associated with higher overall complications and the development of aesthetic deformities. Further prospective studies are warranted to identify surgical and patient factors in determining ideal placement.

## DISCLOSURES

The authors have no financial interest to declare in relation to the content of this article. There are no commercial associations that might pose or create a conflict of interest with information presented in this article, such as consultancies, stock ownership, or patent licensing arrangements. All sources of funds supporting the completion of this study are under the auspices of the University of California Los Angeles. Research reported in this publication was supported by the National Center for Advancing Translational Science of the National Institutes of Health under the UCLA Clinical and Translational Science Institute grant number UL1TR001881.
